# Supplementary material for: Complexome profiling of the Chlamydomonas psb28 mutant reveals TEF5 as an early PSII assembly factor
Source: Plant Cell. 2025 Mar 17;37(6):koaf055. doi: 10.1093/plcell/koaf055 (PMC12164590; doi:10.1093/plcell/koaf055)
Supplement: koaf055_Supplementary_Data [file koaf055_supplementary_data.zip › psb28_interactive.zip!/plots/intensity/qualityPlots/peptides/CYB1_Cre16.g659350.t1.2.html]

### Cre16.g659350.t1.2

Trivial name: CYB1  
SeqCoverage [median]: 23.70  
Mass: 15.01 kDa  
  
 

| Sequence | MissedCleavage | Mass [Da] | wtIntensity | muIntensity | Score |
| --- | --- | --- | --- | --- | --- |
| DATQDFEEIGHSNSAK | 0 | 1747.76 | 1053.28 | 5728.70 | 145.86 |
| YVIGEFEGGDSAPAVAK | 0 | 1708.83 | 4432.99 | 9772.78 | 208.27 |
|  |  |  | 5486.3 | 15501.5 |  |
